# Sociodemographic Patterns in Mood and Anxiety Disorders Among Youth and Young Adults in Canada: An Analysis of the 2015-2021 Surveillance Data

**DOI:** 10.7759/cureus.92238

**Published:** 2025-09-13

**Authors:** Amaka S Odega, Joy Ugwuanyi, Ron Dieba, Oluwatomiwa S Fasoro, Oluwagbeminiyi M Adepeko, Ifeyinwa H Ofuase-Lasekan, Okelue E Okobi

**Affiliations:** 1 Psychiatry, Sault Area Hospital, Sault Ste. Marie, CAN; 2 Psychiatry, NOSM University, Sault Ste. Marie, CAN; 3 Psychiatry and Behavioral Sciences, University of Nigeria Teaching Hospital, Enugu, NGA; 4 Internal Medicine, University of Nigeria Teaching Hospital, Enugu, NGA; 5 Department of Medicine, AdventHealth, Chicago, USA; 6 Clinical Sciences, College of Health Sciences at Obafemi Awolowo University, Ile-Ife, NGA; 7 Family Medicine, Provincial Health Services Authority, Vancouver, CAN; 8 Family Medicine, Larkin Community Hospital Palm Springs Campus, Miami, USA

**Keywords:** anxiety, canada, cchs, epidemiology, mood disorders, public health, sociodemographic disparities, youth mental health

## Abstract

Background and objective

Mood and anxiety disorders are increasingly affecting youth and young adults in Canada, with significant sociodemographic disparities. Understanding these patterns is essential for guiding targeted mental health interventions. Hence, this study aimed to describe the trends in diagnosed mood and anxiety disorders among Canadian youth (12-29 years) from 2015 to 2021, and to compare prevalence across sociodemographic groups (sex, age, income, geography, and ethnocultural identity).

Methods

This study used cross-sectional surveillance data from the Canadian Community Health Survey (2015-2021), analyzing weighted prevalence estimates and 95% confidence intervals (CI) stratified by key sociodemographic factors using descriptive statistical methods.

Results

The overall prevalence of diagnosed mood and anxiety disorders increased from 12.9% (95% CI: 11.7-14.2) in 2015 to 17.3% (95% CI: 15.6-18.9) in 2021. Female youth consistently reported higher rates than males (22.8% vs. 12.0% in 2021). Young adults aged 18-25 had the highest burden compared to adolescents (13.4%) and older youth. The prevalence was disproportionately higher among indigenous populations (33.7%), low-income groups, and residents of Atlantic provinces. Immigrants and racialized groups reported lower prevalence, but this may be attributed to underdiagnosis or systemic barriers to care.

Conclusions

Mood and anxiety disorders among Canadian youth are on the rise, with significant disparities in terms of sex, age, income, and ethnicity. These findings underscore the urgent need for equitable, culturally competent mental health services, early intervention, and policy responses tailored to vulnerable groups. Population-level surveillance combined with clinical insights is crucial to inform targeted mental health strategies and promote resilience in Canada’s youth and young adult populations.

## Introduction

Mood and anxiety disorders are among the most common mental health conditions affecting youth and young adults, often emerging during adolescence and early adulthood, a critical period of emotional, social, and cognitive development [1]. These disorders can impair functioning in school, relationships, and work, and if unaddressed, may lead to long-term psychological and physical health issues [2]. Sociodemographic factors such as age, sex, income, geographic location, and ethnicity are known factors that impact the risk, diagnosis, and management of mental health conditions [3]. Understanding how these factors shape patterns in various mental health disorders is essential for creating equitable and effective policies [4]. 

From 1990 to 2021, anxiety disorders among individuals aged 10-24 spiked by 52%, especially since the emergence of the COVID-19 pandemic in late 2019. Females aged 10-14 years and regions with a middle socio-demographic index (SDI) witnessed the highest rates, and bullying was a key risk factor [5]. Anxiety disorders affect 301 million people globally, or 4.05% of the population, with a 55% rise from 1990 to 2019 [6]. Rates of prevalence, incidence, and disability-adjusted life years (DALYs) continue to increase. Portugal reports the highest prevalence, followed by Brazil, Iran, and New Zealand [7]. 

From a pathophysiological perspective, mood and anxiety disorders are characterized by complex interactions between biological, psychological, and environmental factors [8]. Neurochemical imbalances, particularly involving serotonin, dopamine, and norepinephrine, play a central role in mood regulation [9]. Dysregulation of the hypothalamic-pituitary-adrenal (HPA) axis, genetic vulnerabilities, adverse childhood experiences, and persistent psychosocial stressors further contribute to the development and persistence of these disorders [10]. In youth and young adults, ongoing neurodevelopment in areas responsible for emotional regulation and executive function increases susceptibility. The interaction of these neurobiological mechanisms with adverse life experiences can precipitate or exacerbate mental health conditions during this formative period [11]. 

The current study utilizes data from the Public Health Agency of Canada’s Mental Health Surveillance Tool. This platform provides population-level estimates derived from national health surveys, including the Canadian Community Health Survey (CCHS) [12]. It offers age- and sex-specific estimates and allows stratification by income, region, immigrant status, and ethnicity. The use of such a comprehensive and nationally representative data source enhances the reliability and generalizability of the findings. This study aimed to describe trends in the prevalence of diagnosed mood and anxiety disorders among Canadian youth and young adults using national surveillance data. Specifically, we compared prevalence across key sociodemographic groups (sex, age, income, geography, and ethnocultural identity). These stratifiers were chosen because they represent important social determinants of mental health and are routinely collected in CCHS, enabling equity-focused surveillance to identify groups at higher risk.

## Materials and methods

Data source and study design 

This study employed a cross-sectional, population-based design using data available through the Public Health Agency of Canada’s Mental Health Data Tool. The surveillance estimates are derived from the CCHS, an annual national survey administered by Statistics Canada [12]. The CCHS collects information on health status, healthcare utilization, and health determinants among Canadians aged 12 and above, ensuring wide demographic and regional coverage. The current analysis focused on survey cycles from 2015 to 2021, aiming to track trends and disparities in self-reported diagnosed mood and anxiety disorders among youth and young adults. 

Study participants and questionnaires

The study population included youth and young adults aged 12 to 29 years, with data pooled across multiple annual CCHS cycles. All respondents completed the standardized, interviewer-administered questionnaires either in person or via telephone. Mood and anxiety disorders were identified based on responses to the question: “Do you have dysthymia?” and “Do you have an anxiety disorder such as a phobia, obsessive-compulsive disorder, or a panic disorder?” Only individuals who reported a diagnosis made by a healthcare professional were included in the analysis. The CCHS uses complex multistage sampling to ensure representativeness across Canada’s provinces and territories. 

Data collection and quality assurance 

Data collection was conducted using a robust, standardized protocol developed by Statistics Canada, incorporating both telephone and face-to-face interviews, depending on participant accessibility and survey cycle. Quality assurance measures included interviewer training, ongoing supervision, and verification of responses for consistency. Data weighting was applied to adjust for survey design, non-response, and post-stratification, ensuring that the estimates reflect the Canadian population. As only frequency (percentage) data were available and raw individual-level counts could not be accessed, values are reported as percentages where applicable.

Variables of interest 

The primary outcome variable was the self-reported prevalence of diagnosed mood and/or anxiety disorders. Independent variables included demographic and sociodemographic factors: age group (12-17, 18-25, 26-29), sex (male, female), geographic region (province/territory), household income quintile, immigration status (immigrant or non-immigrant), place of residence (urban vs rural), and ethnocultural group (e.g., White, First Nations off-reserve, Black, South Asian, East/Southeast Asian, etc.). These variables were selected based on their relevance to social determinants of mental health and availability within the CCHS dataset. 

Data analysis and statistical methods 

Descriptive statistics were used to present annual prevalence estimates, along with corresponding 95% confidence intervals (CIs), for all variables of interest. Trends over time were visually examined, and group comparisons were made to detect disparities. All reported prevalence values pertain to the 12-29-year population and were analyzed using survey weights provided by Statistics Canada to ensure national representativeness. Data were analyzed using paired t-tests for two-group comparisons (e.g., gender, age groups, residence, income, and immigrant status) and one-way ANOVA for multi-group comparisons (e.g., geographic regions and ethnocultural groups). Statistical significance was set at p<0.05. All analyses were conducted in IBM SPSS Statistics version 27 (IBM Corp., Armonk, NY), with t/F values and p-values reported accordingly. 

Trends were visually inspected using year-by-year weighted prevalence and 95% CIs. We did not fit time-series regression models to the aggregated prevalence estimates because only yearly aggregated, weighted estimates (rather than individual-level microdata) were available. If microdata become available, appropriate extensions could include survey-weighted logistic regression with year treated as a continuous variable (to estimate average annual change), joinpoint regression to detect changes in trend slope, or generalized estimating equations (GEE) accounting for intra-cluster correlation across survey cycles.

Ethical considerations 

This study is based entirely on secondary, publicly available, de-identified data from the CCHS and the Public Health Agency of Canada. Hence, ethics approval was not required. The data used complies with all national privacy and data protection regulations. All results were reported in aggregate form, with no individual-level identifiers, ensuring participant anonymity and confidentiality throughout the analysis.

## Results

This section presents the prevalence of mood and anxiety disorders among Canadian youth and young adults between 2015 and 2021. The findings are reported across various sociodemographic categories, including gender, age, place of residence, income, geographic region, immigrant status, and ethnocultural background. All values are presented as percentages with their corresponding 95% confidence intervals (CI), formatted as (95% CI: LL-UL). This section focuses on describing the data trends observed over time without offering interpretation. 

Overall prevalence 

Among the total population aged 12 and above, the prevalence of mood and anxiety disorders increased consistently over the study period. In 2015, the prevalence was 12.9% (95% CI: 11.7-14.2), which rose steadily to 17.3% (95% CI: 15.6-18.9) in 2021. The average prevalence across the seven years was 15.1% (95% CI: 13.7-16.5). A notable year-on-year increase was observed between 2017 (14.0%; 95% CI: 12.7-15.2) and 2019 (16.7%; 95% CI: 15.4-18.1). A minor dip occurred in 2020 (15.6%; 95% CI: 13.9-17.3), followed by another rise in 2021. Table 1 presents the distribution of mood and anxiety disorders among Canadian youth and young adults by gender, age, income, region, residence, immigration status, and ethnocultural identity from 2015 to 2021. 

**Table 1:** Sociodemographic distribution of mood and anxiety disorders among Canadian youth and young adults

Category		2015	2016	2017	2018	2019	2020	2021	P value
Overall	Total population	12.9 (11.7-14.2)	13.6 (12.4-14.8)	14.0 (12.7-15.2)	15.5 (14.1-16.8)	16.7 (15.4-18.1)	15.6 (13.9-17.3)	17.3 (15.6-18.9)	
Gender	Male	10.1 (8.5-11.6)	10.4 (8.8-12)	9.8 (8.4-11.1)	11.2 (9.5-12.8)	11.8 (10.2-13.4)	11.4 (9.4-13.4)	12 (9.9-14.1)	t = -12.62, p < 0.00001
	Female	16.1 (14.1-18)	16.9 (15.2-18.7)	18.4 (16.2-20.5)	20.1 (18-22.2)	22.2 (20.1-24.3)	20.4 (17.5-23.4)	22.8 (20.3-25.4)	
Age groups	12-17 years	10.5 (9-12)	9.7 (8.4-11.1)	9.6 (8.4-10.8)	11.8 (10.3-13.3)	12.5 (10.9-14.1)	11.7 (9.9-13.5)	13.4 (11.7-15.1)	t = -14.96, p < 0.00001
	Young adults (18-25 years)	14.4 (12.6-16.3)	16.1 (14.4-17.8)	16.8 (14.9-18.6)	17.7 (15.8-19.7)	19.4 (17.5-21.4)	18.4 (15.7-21)	19.9 (17.4-22.4)	
Place of residence	Population centre	12.9 (11.5-14.3)	14.0 (12.7-15.3)	14.4 (13-15.9)	15.6 (14.1-17.1)	16.8 (15.3-18.3)	15.8 (13.9-17.7)	17.3 (15.4-19.1)	t = 2.25, p = 0.033
	Rural area	13.1 (10.9-15.3)	11.4 (9-13.8)	11.4 (9.5-13.3)	14.9 (12.6-17.1)	16.5 (13.8-19.1)	14.7 (11.4-18.1)	17.3 (14.2-20.4)	
By household income quintile	Q1 (lowest)	14.9 (12.5-17.4)	15.4 (13.2-17.7)	16.0 (13.7-18.3)	19.0 (16.1-21.9)	19.4 (16.5-22.2)	18.7 (14.8-22.7)	18.2 (14.8-21.7)	t = 2.25, p = 0.031
	Q2	15.6 (12-19.3)	14.8 (12.1-17.4)	15.4 (12.6-18.3)	15.8 (12.8-18.8)	15.7 (12.7-18.7)	12.6 (9.6-15.7)	17.4 (13.2-21.5)	
By geographic region	Atlantic provinces	20.9 (17.2-24.6)	19.3 (16-22.5)	20 (16.6-23.5)	21.4 (17.8-25.1)	21.1 (17.3-24.8)	20 (14.3-25.7)	24.1 (20.3-27.9)	F = 17.75, p < 0.000001
	British Columbia (BC)	12.4 (9.4-15.3)	13.5 (10.2-16.7)	15.9 (12.3-19.4)	11.8 (9.1-14.4)	17.2 (13.6-20.8)	17.5 (12.4-22.6)	20.3 (15.3-25.3)	
	Ontario (ON)	15.1 (12.5-17.7)	13.3 (11.3-15.4)	14.6 (12.1-17)	16.4 (13.9-19)	17.2 (14.9-19.5)	16.4 (13.5-19.4)	17.8 (14.8-20.9)	
	Prairie provinces	11.9 (9.8-14)	15.2 (13-17.4)	14.2 (12-16.4)	17.2 (14.4-20)	16.9 (14-19.9)	14.5 (11.5-17.6)	18.5 (15.6-21.4)	
	Quebec (QC)	8.1 (6.2-10)	11 (8.7-13.4)	9.8 (8-11.6)	12.5 (10.3-14.6)	14 (11.5-16.4)	12.6 (9.3-15.9)	11.1 (8.5-13.8)	
Based on immigrant status	Immigrant	7.4 (3.7-11.0)	6.9 (4.8-9.1)	8.9 (6.1-11.7)	6.2 (3.5-8.9)	8.4 (6.0-10.9)	5.2 (2.8-7.6)	9.9 (6.3-13.5)	t = -9.41, p < 0.0001
	Non-immigrant	14.2 (12.8-15.5)	14.7 (13.4-15.9)	15.2 (13.8-16.6)	18.0 (16.5-19.5)	19.0 (17.4-20.5)	18.6 (16.6-20.6)	19.2 (17.3-21.1)	
By ethnocultural group	Racialized individuals	8.1 (5.4-10.8)	6.4 (4.8-7.9)	9.5 (6.8-12.1)	8.2 (6.0-10.4)	10.3 (8.0-12.5)	7.1 (4.3-9.9)	9.9 (7.1-12.6)	F = 84.91, p < 0.000001
	First Nations off-reserve/Inuit/Métis	23.1 (16.9-29.3)	27.7 (21.5-33.9)	25.9 (19.5-32.4)	26.1 (20.8-31.3)	33.7 (27.6-39.9)	27.9 (20.7-35.1)	33.7 (26.1-41.0)	
	Arab/West Asian	-	-	9.4 (3.3-15.6)	-	-	-	18.1 (6.4-29.8)	
	Black		10.1 (4.8-15.4)			14.1 (7.8-20.3)		11.2 (3.7-18.7)	
	East/Southeast Asian	-	2.9 (1.2-4.6)	6.8 (3.7-9.9)	6.5 (2.3-10.6)	7.7 (4.4-11)	-	7.4 (3.2-11.5)	
	Other/multiple origins	10.9 (6-15.8)	10.8 (6.8-14.8)	19.6 (9.6-29.6)	14.0 (8.1-20)	24.5 (7.9-41)			
	South Asian		3.5 (1.1-5.8)	8.4 (3.8-12.9)		9.5 (4.6-14.4)	8.1 (2.9-13.3)	7.7 (2.7-12.8)	
	White	14.3 (12.9-15.7)	15 (13.6-16.4)	15.1 (13.7-16.5)	18.4 (16.6-20.1)	19 (17.3-20.7)	19.1 (16.9-21.4)	20.5 (18.3-22.6)	

Prevalence by gender 

**Figure 1:**
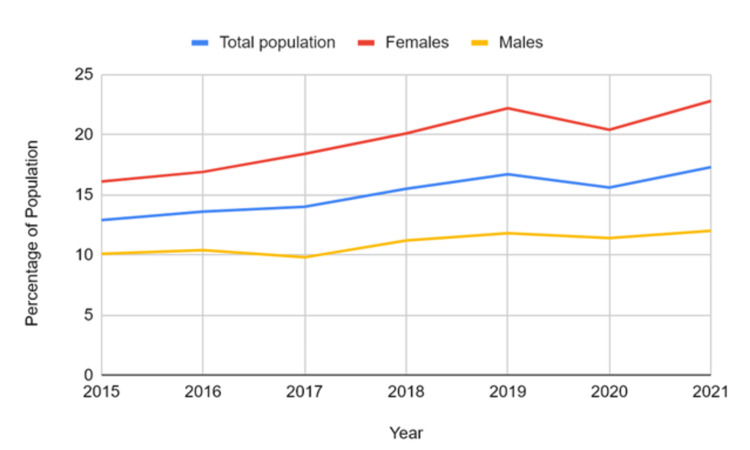
Overall and gender-wise trends in mood and anxiety disorders among Canadian youth and young adults

A consistent gender gap in prevalence was observed throughout the years. Males reported lower rates of mood and anxiety disorders, ranging from 9.8% in 2017 (95% CI: 8.4-11.1) to 12.0% in 2021 (95% CI: 9.9-14.1). In contrast, females exhibited higher and steadily increasing rates, from 16.1% in 2015 (95% CI: 14.1-18.0) to 22.8% in 2021 (95% CI: 20.3-25.4). This gender difference remained consistent throughout the period, with females showing prevalence levels nearly twice as high as males by the end of the study window. A paired t-test comparing males and females showed a highly significant difference (t = -12.62, p < 0.00001), indicating that females consistently had higher values compared to males. A paired t-test comparing males and females showed a highly significant difference (t = -12.62, p < 0.00001), indicating that females consistently had higher values compared to males. Figure 1 displays mood and anxiety disorder rates by overall and gender, highlighting consistently higher prevalence in females than males among Canadian youth and young adults.

Prevalence by age group 

**Figure 2:**
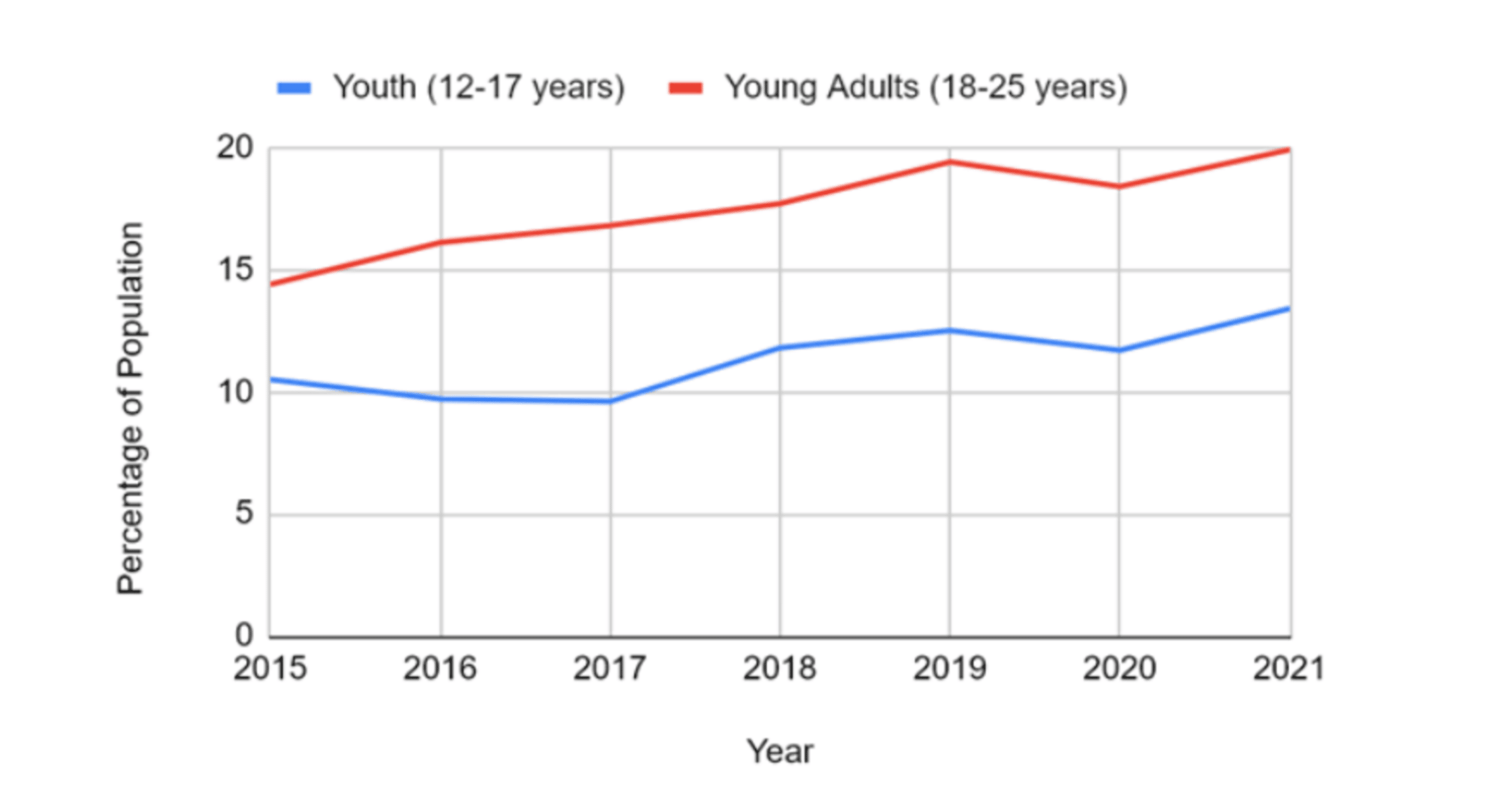
Prevalence of mood and anxiety disorders by age group among Canadian youth and young adults

The data showed that young adults aged 18-25 had a higher prevalence of mood and anxiety disorders than adolescents aged 12-17. Among adolescents, rates increased from 10.5% in 2015 (95% CI: 9.0-12.0) to 13.4% in 2021 (95% CI: 11.7-15.1). Young adults reported a rate of 14.4% in 2015 (95% CI: 12.6-16.3), which rose to 19.9% in 2021 (95% CI: 17.4-22.4). Both age groups experienced an upward trend, with young adults consistently demonstrating a higher prevalence across all years. When comparing 12-17 years vs. young adults (18-25 years), the difference was also highly significant (t = -14.96, p < 0.00001), with young adults demonstrating notably higher values than adolescents. Figure 2 shows that mood and anxiety disorders were most prevalent among youth aged 15-24, with older adolescents consistently reporting the highest burden across the surveillance period. 

Prevalence by place of residence 

**Figure 3:**
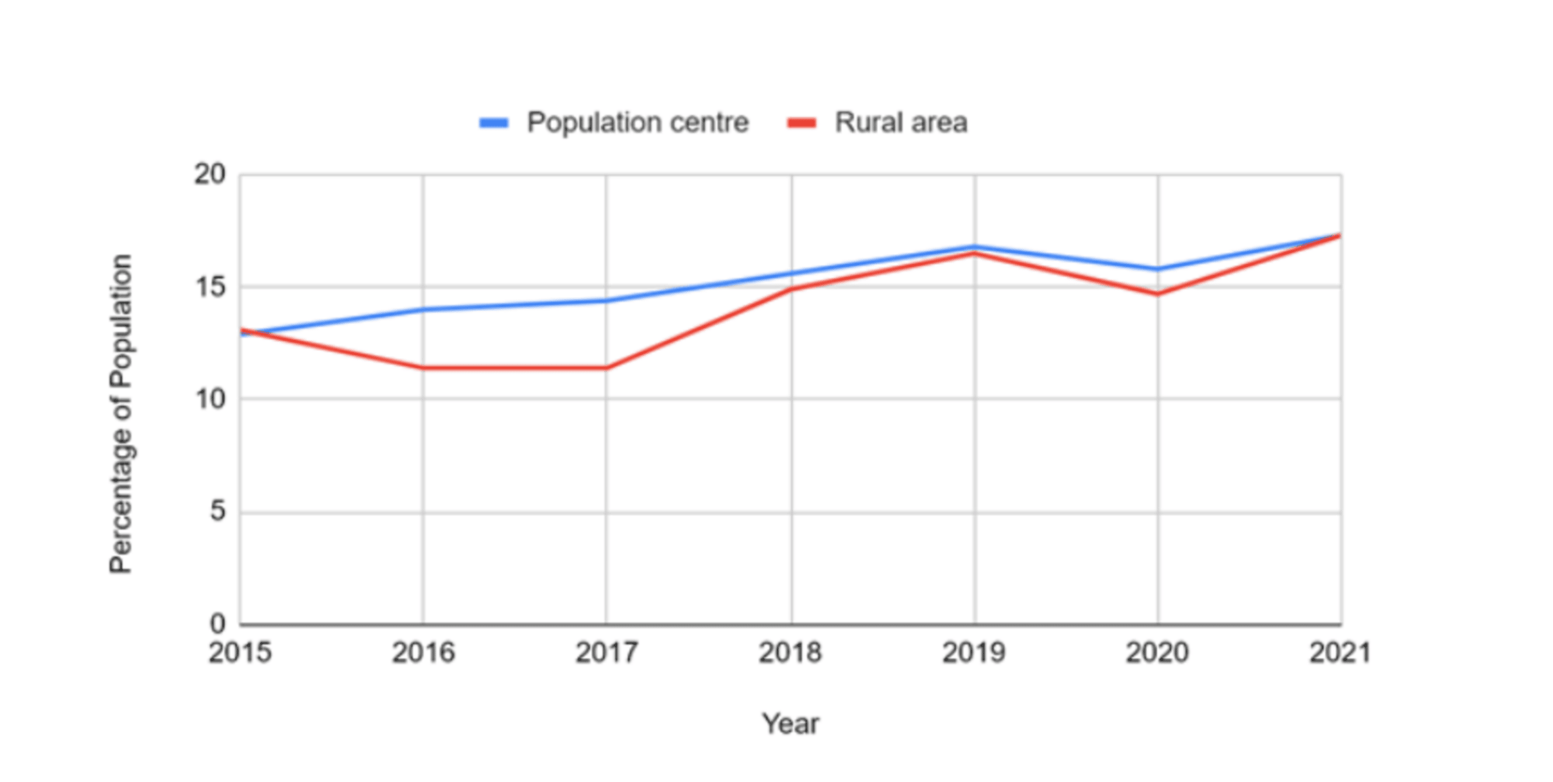
Urban-rural differences in mood and anxiety disorders in Canadian youth and young adults

Individuals living in population centres and rural areas followed similar upward trends over the seven years. In urban areas, the prevalence increased from 12.9% in 2015 (95% CI: 11.5-14.3) to 17.3% in 2021 (95% CI: 15.4-19.1). Rural areas started at 13.1% (95% CI: 10.9-15.3), dipped in 2016 and 2017 to 11.4% (95% CI: 9.0-13.8 and 9.5-13.3, respectively), and then rose again to 17.3% (95% CI: 14.2-20.4) in 2021. For population centres vs. rural areas, there was a statistically significant difference (t = 2.25, p = 0.033), suggesting slight but meaningful variation between urban and rural residents. Figure 3 shows that while urban residents generally exhibited slightly higher prevalence rates of mood and anxiety disorders than rural youth, this pattern was not consistent across all years. This suggests fluctuations in prevalence rather than a consistent urban-rural disparity. 

Prevalence by household income quintile 

**Figure 4:**
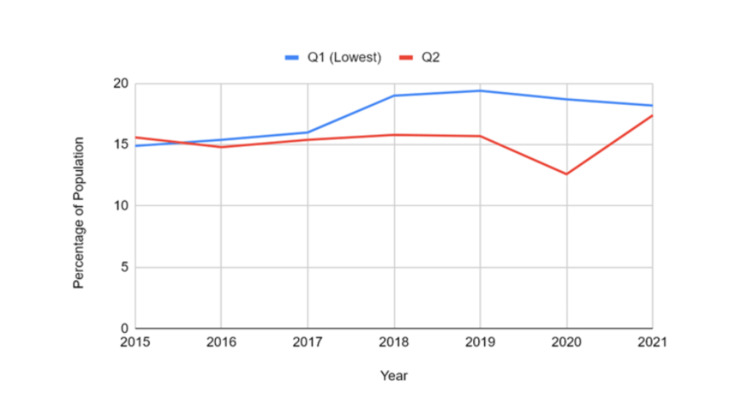
Mood and anxiety disorder prevalence by household income quintile in Canadian youth and young adults

Income-based disparities were evident throughout the data. Those in the lowest income quintile (Q1) experienced a rise from 14.9% in 2015 (95% CI: 12.5-17.4) to 18.2% in 2021 (95% CI: 14.8-21.7). Individuals in the second quintile (Q2) reported rates of 15.6% in 2015 (95% CI: 12.0-19.3), with fluctuations over the years and a peak of 17.4% in 2021 (95% CI: 13.2-21.5). These patterns reveal a higher prevalence among lower-income groups compared to the general population. Comparing the lowest Q1 with the Q2 revealed a significant difference (t = 2.25, p = 0.031), indicating that income level influenced the observed values. Figure 4 shows a clear socioeconomic gradient, with the lowest income quintile experiencing the highest prevalence, indicating strong links between income-related stress and mental health. 

Prevalence by geographic region 

**Figure 5:**
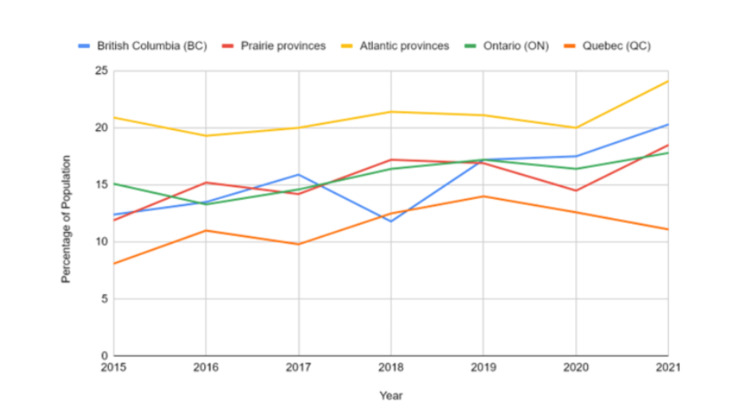
Regional patterns of mood and anxiety disorders across Canadian provinces and territories

Provincial-level data highlighted regional variation in prevalence trends. The Atlantic provinces consistently reported the highest levels, starting at 20.9% in 2015 (95% CI: 17.2-24.6) and reaching 24.1% in 2021 (95% CI: 20.3-27.9). British Columbia followed with a rise from 12.4% (95% CI: 9.4-15.3) in 2015 to 20.3% (95% CI: 15.3-25.3) in 2021. Ontario’s prevalence increased from 15.1% (95% CI: 12.5-17.7) to 17.8% (95% CI: 14.8-20.9), while the Prairie provinces rose from 11.9% (95% CI: 9.8-14.0) to 18.5% (95% CI: 15.6-21.4). Quebec maintained lower values throughout, starting at 8.1% in 2015 (95% CI: 6.2-10.0) and reaching 11.1% in 2021 (95% CI: 8.5-13.8). An ANOVA across Atlantic provinces, British Columbia, Ontario, Prairie provinces, and Quebec showed a strong regional variation (F = 17.75, p < 0.000001), highlighting that geographic location significantly affected the results. Figure 5 shows that youth in British Columbia and Atlantic provinces had higher disorder rates, while Prairie and Northern regions reported comparatively lower prevalence levels.

Prevalence by immigrant status 

**Figure 6:**
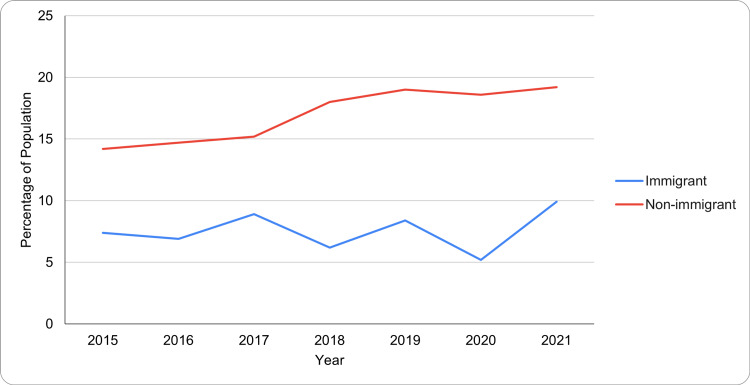
Mood and anxiety disorders by immigrant status among Canadian youth and young adults

A strong difference was observed between immigrant and non-immigrant populations. Immigrants had significantly lower prevalence rates, ranging from 5.2% in 2020 (95% CI: 2.8-7.6) to 9.9% in 2021 (95% CI: 6.3-13.5). Non-immigrants, on the other hand, showed a steady increase from 14.2% in 2015 (95% CI: 12.8-15.5) to 19.2% in 2021 (95% CI: 17.3-21.1). A paired t-test between immigrants and non-immigrants found a highly significant difference (t = -9.41, p < 0.0001), with non-immigrants exhibiting higher values than immigrants. Figure 6 shows a higher prevalence among non-immigrant youth compared to immigrants, potentially reflecting protective cultural factors or reduced reporting among immigrant populations. 

Prevalence by ethnocultural group 

Ethnocultural group-level differences were prominent. First Nations, Inuit, and Métis individuals living off reserve reported the highest rates across all years, increasing from 23.1% in 2015 (95% CI: 16.9-29.3) to 33.7% in both 2019 and 2021 (95% CI: 27.6-39.9 and 26.1-41.3, respectively). Racialized individuals had a comparatively lower prevalence, ranging from 6.4% in 2016 (95% CI: 4.8-7.9) to 10.3% in 2019 (95% CI: 8.0-12.5). White individuals reflected the national average trend, rising from 14.3% in 2015 (95% CI: 12.9 -- 15.7) to 20.5% in 2021 (95% CI: 18.3-22.6). 

More detailed categorization revealed additional variation. East/Southeast Asian individuals had the lowest reported rates, with values such as 2.9% in 2016 (95% CI: 1.2-4.6) and 7.7% in 2019 (95% CI: 4.4-11.0). South Asians ranged between 3.5% in 2016 (95% CI: 1.1-5.8) and 9.5% in 2019 (95% CI: 4.6-14.4). The Arab/West Asian group had rates of 9.4% in 2017 (95% CI: 3.3-15.6) and 18.1% in 2021 (95% CI: 6.4-29.8). Black individuals reported between 10.1% in 2016 (95% CI: 4.8-15.4) and 14.1% in 2019 (95% CI: 7.8-20.3). A one-way ANOVA across racialized individuals, First Nations off reserve/Inuit/Métis, White, and other ethnocultural groups showed the largest variation among all categories (F = 84.91, p < 0.000001), confirming substantial differences by ethnocultural identity. The “Other/Multiple origins” category showed highly variable estimates, including a peak of 24.5% in 2019 (95% CI: 7.9-41.0), reflecting heterogeneity. Notably, First Nations, Inuit, and Métis individuals consistently reported the highest rates across all categories and years.

## Discussion

Mood and anxiety disorders remain a growing concern in Canadian public health, particularly among youth and young adults aged 12 to 29 years. The findings from this analysis of 2015-2021 surveillance data highlight a consistent upward trend in the prevalence of diagnosed mood and anxiety disorders across this population. Several sociodemographic subgroups, including females, young adults aged 18-25, individuals from low-income households, and Indigenous youths, were disproportionately affected. This discussion integrates the current findings with clinical and molecular insights, presents a representative clinical case scenario, explores pathophysiological mechanisms, compares data with other literature, and considers public health and policy implications in the context of Canadian and U.S. guidelines. 

The lower reported rates in immigrant populations when compared with non-immigrant populations could be attributed to language barriers, unfamiliar healthcare system, or non-disclosure of symptoms to avoid stigma [3,12]. To illustrate the clinical relevance of these findings, consider the following case: A 19-year-old female university student from Nova Scotia presented to her primary care physician with persistent low mood, anxiety, disrupted sleep, and difficulty concentrating for over six months. She reported past adverse childhood experiences and recent academic stress. On screening with the PHQ-9 and GAD-7, her scores were in the moderate to severe range. She was diagnosed with major depressive disorder and generalized anxiety disorder. This scenario reflects the broader pattern observed in our data: females and young adults exhibit the highest burden of mood and anxiety disorders. Early adulthood represents a particularly vulnerable period, marked by transitions in social roles, exposure to academic and occupational stress, and increasing independence. Literature supports this trend: a Canadian study by Wiens et al. found that nearly 25% of postsecondary students met criteria for a mood or anxiety disorder, with significantly higher rates among females [13]. 

Although mood and anxiety disorders are not age-specific, young adults (18-25) are more psychologically vulnerable. Peculiar life transitions, including exiting adolescence to begin adulthood, play a role in amplifying this vulnerability [4,11]. Self-identity discovery, deciding on crucial life issues, and achieving financial autonomy are some major challenges that put additional strains on all areas of their health, particularly mental health. Research shows that early childhood trauma negatively affects certain hormones (e.g., cortisol) and brain regions (e.g., hippocampus, prefrontal cortex) responsible for emotion regulation in the body [10,11]. These effects typically present around this age, thereby increasing their susceptibility to mood and anxiety disorders. 

Mood and anxiety disorders are heterogeneous in etiology and presentation, but several core pathophysiological mechanisms are well established. Neurobiologically, these disorders are linked to functional changes in limbic and prefrontal brain circuits, particularly the amygdala, hippocampus, and anterior cingulate cortex. Chronic stress leads to dysregulation of the HPA axis, resulting in prolonged cortisol secretion, which in turn affects neuroplasticity and emotional regulation. Studies have shown that early-life adversity, common among populations in lower-income brackets and certain racialized groups, can permanently alter HPA responsiveness, increasing susceptibility to psychopathology. 

In mood disorders such as major depression, reductions in hippocampal volume and prefrontal cortex activity are common findings on neuroimaging. In anxiety disorders, increased amygdala activation is typically observed in response to perceived threats. These abnormalities lead to heightened emotional reactivity and impaired cognitive control, key features of the clinical syndromes [10,14]. At the molecular level, the monoamine hypothesis remains foundational. Alterations in neurotransmitter levels, serotonin (5-HT), norepinephrine (NE), and dopamine (DA), disrupt mood regulation, reward processing, and arousal. Selective serotonin reuptake inhibitors (SSRIs), which increase synaptic 5-HT availability, remain the first-line pharmacologic treatment, highlighting the relevance of this pathway [9-10]. 

Additionally, neuroinflammation is increasingly implicated in the pathogenesis of both mood and anxiety disorders. Elevated levels of pro-inflammatory cytokines (e.g., IL-6, TNF-α) have been identified in patients with depression, correlating with symptom severity. These markers are thought to impair neurogenesis and promote oxidative stress, particularly in genetically vulnerable individuals. Epigenetic factors such as methylation of the BDNF (brain-derived neurotrophic factor) gene have also been associated with both early trauma and mood disorder onset [15]. 

The present study’s findings align with national and international literature. For instance, the Canadian Community Health Survey - Mental Health 2012 reported a 12-month prevalence of mood disorders at 7.1% among Canadians aged 15+, with anxiety disorders at 8.6%. Our data suggest these numbers have increased significantly in youth, with rates exceeding 20% among females by 2021 [16]. A study in the U.S. using National Survey on Drug Use and Health (NSDUH) data revealed similar patterns: depression among U.S. young adults increased from 8.1% in 2008 to 15.8% in 2019. Canadian trends are comparable, especially in post-pandemic years, indicating global shifts in youth mental health [17]. Notably, Indigenous youth in our study had the highest prevalence rates (e.g., 33.7% in 2021), echoing findings by Bombay et al., who documented high psychological distress among First Nations, Métis, and Inuit youth. Historical trauma, systemic discrimination, and limited access to culturally appropriate care are significantly associated with these disparities [18]. 

Our data show that female youth reported nearly twice the prevalence of mood and anxiety disorders compared to males by 2021 (22.8% vs. 12%). This sex difference is well documented and may relate to hormonal fluctuations, greater interpersonal stress exposure, and gender-specific coping mechanisms. Hormonal differences and significant structural and functional brain differences between men and women have been explored as additional factors causing variations in anxiety. This involves the prefrontal cortex, hippocampus, and the extended amygdala complex [19], and the hypothesized hormonal factors with added periodic fluctuations across reproductive phases, linked to changes in anxiety symptom severity. 

Some studies have suggested that a key metabolite of progesterone, allopregnanolone, acts as a potent positive allosteric modulator of GABAA receptors (a predominant inhibitory neurotransmitter system). As observed in animal models, dysregulation in this system, such as decreased inhibition of GABA receptors during periods of low progesterone (and thus a relative decrease in allopregnanolone), can increase the excitability of panic circuitry in the brain's periaqueductal gray (PAG) region. Conversely, high levels of allopregnanolone provide anxiolytic and antidepressant effects [20]. Meanwhile, a recent study done on mouse models provides potential evidence that the absence of testosterone results in the pattern of stress response commonly observed in the high trend of anxiety among females, suggesting it’s possible anxiolytic influence on brain activity, specifically in the amygdala, a key region for stress activation. Mice that had never been exposed to testosterone (both male and female) showed unusually high levels of amygdala activity when placed in proximity with unfamiliar mice, due to an exaggerated threat signal. In contrast, mice exposed to testosterone showed no increase in this neural activity, thereby suggesting that testosterone actively reshapes the brain circuitry to reduce exaggerated responses to social stress, thus potentially offering a protective effect against anxiety [21]. 

Income was another key determinant. Youth in the lowest income quintile consistently reported the highest prevalence (e.g., 18.2% in 2021), reinforcing the socioeconomic gradient in mental health. Financial stress, food insecurity, and limited access to mental health services disproportionately affect low-income households [5]. Kirkbride et al. highlight that financial stress and life insecurity, key social determinants, exacerbate mental health challenges by creating chronic psychosocial stressors [3]. These stressors, such as inability to meet basic needs or unstable living conditions, can dysregulate the hypothalamic-pituitary-adrenal axis, increasing cortisol levels and heightening vulnerability to mood and anxiety disorders. Moreover, low income is strongly associated with higher rates of mental health issues, as evidenced by studies like Bie et al., which found a prevalence of mood and anxiety disorders of 18.2% in the lowest income quintile among Canadian youth compared to the national average of 17.3% [5]. Low-income individuals often face additional barriers, like limited access to mental health services, further compounding life insecurity and worsening psychological outcomes.

Geographically, the Atlantic provinces recorded the highest rates (e.g., 24.1% in 2021). Possible explanations include socioeconomic disadvantages, rurality, and service shortages. Urban-rural trends converged over time, but early in the dataset, urban youth had a slightly higher prevalence, potentially linked to environmental stressors like overcrowding and lower community cohesion. Clinically, these findings emphasize the need for early detection and intervention, as well as ensuring gender-specific considerations in management guidelines. Routine mental health screening in schools and primary care settings can help identify high-risk youth. Integrating mental health into school curricula and enhancing digital access to therapy (e.g., iCBT, telepsychiatry) are promising strategies. The Canadian Pediatric Society (CPS) recommends regular mental health assessments for adolescents, especially those with risk factors such as trauma, substance use, or academic decline [22]. 

From a policy perspective, the Canadian Mental Health Strategy and Wellness Together Canada platform have expanded access to mental health supports. However, gaps remain, particularly for Indigenous and rural populations. Culturally safe care, improved funding for community-based programs, and expansion of youth-specific mental health clinics are urgently needed [23]. In the U.S., the Youth Mental Health Protection Act and 988 Crisis Line reflect growing recognition of youth mental health. Cross-country collaboration may help develop standardized, equity-oriented interventions. 

Given the limitations of self-reported data and diagnostic variability, future research should incorporate clinical diagnostic tools and longitudinal designs to assess causality. There is also a need for granular data on intersecting identities (e.g., LGBTQ+ youth, neurodiverse populations) who may face compounded vulnerabilities. Additionally, exploring resilience factors, such as social support, physical activity, and access to green spaces, could inform strength-based approaches to mental health promotion [24]. Preventive interventions tailored to the social realities of youth, such as anti-bullying programs, social media literacy, and employment support, are critical. Investments in upstream determinants of health, including housing, education, gender equity, and income security, will have overall downstream effects on youth mental health [25]. 

The lower reported rates in the immigrant population, while positive, may not mean that immigrants truly have lower mental disorder rates. Instead, as Kirkbride and colleagues discussed, social factors like language barriers, limited access to culturally appropriate care, and stigma around mental illness in some communities may make immigrants less likely to seek help or receive a diagnosis. So, the lower reported rates could reflect the complexities surrounding the experience and expression of mental health issues across different cultural backgrounds [3,12] 

Strengths and limitations 

One of the primary strengths of this study is its use of a large, nationally representative dataset from the CCHS, enabling population-level analysis across all provinces and territories. The inclusion of data over eight years allows for robust trend analysis and identification of evolving mental health patterns. The ability to stratify data by multiple sociodemographic variables, such as age, sex, income, immigration status, and ethnocultural background, provides a granular view of disparities in mental health prevalence. Additionally, the study aligns epidemiological findings with pathophysiological and molecular insights, enriching the public health interpretation with clinical relevance.

Despite its strengths, the study has several limitations that must be acknowledged. First, the data are based on self-reported diagnoses, which may be subject to recall bias or underreporting, especially among marginalized or underserved groups who may lack access to formal healthcare. Second, the cross-sectional design does not allow for causal inference or assessment of incidence over time. Third, mental health conditions are complex and may vary in severity, comorbidity, and duration, factors not captured in the survey. Moreover, some estimates, particularly among smaller population subgroups (e.g., ethnocultural minorities), carry high variability and should be interpreted with caution. Lastly, the data do not account for undiagnosed cases, informal care use, or digital mental health interventions, potentially underestimating the true burden. In addition, because only aggregated, weighted prevalence estimates were available (individual-level CCHS records were not accessed), multivariable-adjusted analyses to control for potential confounding could not be performed; this lack of multivariable adjustment is an important limitation. Future studies using longitudinal and clinically validated assessments are needed to address these gaps and deepen our understanding of youth mental health in Canada.

## Conclusions

This study provides a comprehensive overview of mood and anxiety disorder trends among Canadian youth and young adults aged 12-29 between 2015 and 2021. The results reveal a consistent rise in prevalence, particularly among females, young adults, low-income individuals, and Indigenous populations. These findings emphasize the significant burden of mental health conditions within sociodemographically vulnerable groups and call for targeted, equitable mental health services and prevention strategies. Integrating epidemiological surveillance with medical and molecular insights will ensure that this research contributes a nuanced understanding of the biopsychosocial dynamics driving mental illness in young Canadians. Early intervention, culturally sensitive care, and systemic efforts to address social determinants of health are imperative. Going forward, coordinated efforts between healthcare providers, educators, policymakers, and communities will be essential to reverse current trends and support mental wellness across diverse youth populations in Canada.
